# Does Preseason Training Load Shape Body Composition? External Load, Internal Load, and Fatigue in Semi-Professional Football Players

**DOI:** 10.3390/jfmk11040410

**Published:** 2026-10-06

**Authors:** José Carlos Ponce-Bordón, Jorge Polo-Tejada, David Lobo-Triviño, Víctor Toro-Román

**Affiliations:** 1 Faculty of Sport Sciences, University of Extremadura, 10003 Caceres, Spain; jpolote@unex.es (J.P.-T.); davidlt@unex.es (D.L.-T.); 2 Institute for Research and Innovation in Sport, University of Extremadura, 10003 Caceres, Spain; 3 Research Group in Technology Applied to High Performance and Health, Department of Health Sciences, TecnoCampus, Universitat Pompeu Fabra, 08302 Mataró, Spain; vtoro@tecnocampus.cat

**Keywords:** athlete monitoring, bioelectrical impedance analysis, fat-free mass, soccer, wellness

## Abstract

**Background:** Monitoring athletes’ responses to training is essential for optimizing performance and player management in football. Although training load is considered an important component of physiological adaptations, its relationship with body composition changes during preseason remains unclear. **Objectives**: This study aimed to (i) examine changes in body composition, training load, and fatigue throughout the preseason and (ii) determine whether accumulated training load and fatigue were associated with body composition changes in semi-professional football players. **Methods**: Twenty semi-professional male football players from the same team were monitored during a six-week preseason that included 28 training sessions and seven friendly matches. Body composition was assessed using bioelectrical impedance analysis at the beginning (Testing Session 1, TS1), middle (Testing Session 2, TS2), and end (Testing Session 3, TS3) of the preseason. External load and heart rate (HR) were quantified using Global Positioning System (GPS) devices, while internal load was assessed through session rating of perceived exertion (RPE), and fatigue was assessed using wellness questionnaires. **Results**: Significant changes in body composition were observed across the preseason. Fat mass increased, whereas fat-free mass, lean mass, and skeletal muscle mass decreased by TS3 (all *p* < 0.01; large, standardized effect sizes). No significant associations were found between accumulated external load, internal load, HR exposure, fatigue indicators, and body composition changes after baseline adjustment and multiple comparisons. **Conclusions**: Significant alterations in body composition were observed over the preseason period. Given the observational design and absence of a non-training control group, the observed changes cannot be attributed solely to the preseason training stimulus.

## 1. Introduction

Football is an intermittent team sport characterized by high physical demands and repeated high-intensity actions, including sprinting, accelerations (ACC), decelerations (DEC), and changes of direction [1,2]. These actions impose substantial mechanical and metabolic demands, and their accumulation across training sessions and matches may contribute to both physical adaptations and fatigue accumulation throughout the season [3]. Match-analysis research has shown that elite players perform numerous high-intensity efforts interspersed with periods of lower-intensity activity, highlighting the complex metabolic and mechanical demands of the sport [4,5]. Consequently, the preseason period is considered a critical phase within the annual training cycle, as it provides an opportunity to re-establish physical fitness following the off-season and prepare players for the physical and physiological demands of competition [6,7,8].

To optimize training adaptations, coaches regularly monitor training load using both external and internal measures. External load reflects the physical work completed by the athlete and is commonly quantified through Global Positioning System (GPS)-derived variables [9]. In contrast, internal load represents the individual psychophysiological response to the imposed stimulus and may be assessed through subjective and objective measures, including ratings of perceived exertion [10]. Current training models consider both dimensions provide complementary information regarding the training process and suggest that they should be interpreted together to understand athletes’ responses to exercise stimuli [9,10]. In addition, training load monitoring should be complemented by fatigue assessment to better characterize the different responses elicited by training [11]. Fatigue is generally defined as any reduction in physical or mental performance [12,13]. The use of self-report questionnaires to monitor training responses has increased considerably in both applied and research settings [14].

Body composition is another factor associated with football performance [15,16,17]. Excess fat mass (FM) represents a non-functional load that may impair locomotor efficiency and high-intensity performance, whereas greater lean mass (LM) and skeletal muscle mass (SMM) have been associated with greater strength, power, and sprinting ability [18,19]. Previous studies have reported that preseason training may induce favorable adaptations in body composition, including reductions in FM and increases in fat-free mass (FFM), although the magnitude of these changes may vary among players [18,20]. Conversely, the off-season period has been associated with potentially detrimental changes, such as increased body fat and reduced physical performance, emphasizing the importance of appropriate training prescription and monitoring during the preseason [21,22].

The dose–response relationship between training load and physiological adaptation is a central concept in sports science, as accumulated training stimuli may influence changes in fitness, performance, injury risk, and body composition [23,24]. In football, training load indicators have been associated with improvements in aerobic fitness and neuromuscular performance [24,25,26]. However, although preseason training may induce favorable changes in body composition, including reductions in FM and increases in FFM, the extent to which these adaptations are associated with cumulative training load and fatigue-related responses remains unclear [18,20,27]. Importantly, body composition is influenced by multiple interacting factors, including energy intake, total energy expenditure, macronutrient intake, and the type and magnitude of the training stimulus, particularly resistance training. Therefore, external load measures, such as GPS-derived variables, should be considered as indicators of only one component of the overall training stimulus rather than direct determinants of body composition [28,29]. Moreover, most previous studies have focused on external load variables, while the role of internal load and fatigue status in influencing changes in body composition has received less attention [30,31]. Therefore, examining the relationships among external load, internal load, fatigue-related responses, and body composition in semi-professional football players may provide practitioners with useful information to improve training prescription and individualize player management during the preseason.

Similarly, fatigue and wellness responses may provide complementary information regarding how players tolerate and recover from accumulated training stress. While external and internal load quantify the imposed stimulus and the psychophysiological response to training, wellness measures may reflect individual recovery status and factors such as perceived fatigue, sleep quality, muscle soreness, and psychological stress [32]. Persistent fatigue or inadequate recovery may influence an athlete’s capacity to tolerate and perform subsequent training stimuli, potentially affecting the magnitude of adaptations achieved over time. Thus, fatigue-related variables may provide complementary information on individual responses to the accumulated training process and may be associated with changes in body composition rather than serve as direct determinants. Nevertheless, these relationships are likely to be multifactorial and should be interpreted as associations rather than causal effects [33].

Although dual-energy X-ray absorptiometry (DXA) is often considered a reference method for body composition assessment, bioelectrical impedance analysis (BIA) has become widely used in applied football settings due to its practicality, low cost, portability, and non-invasive nature [34,35]. Systematic reviews and methodological studies support the applicability of BIA in athletic populations, particularly when standardized protocols are followed [36,37,38]. Therefore, the aims of the present study were: (i) to compare the evolution of external load, internal load, heart-rate (HR) exposure, fatigue-related variables, and body composition, assessed using BIA, throughout the football preseason period and (ii) to examine the associations between external and internal training load, fatigue-related variables, and changes in body composition throughout the preseason. Based on previous evidence, we hypothesized that favorable changes in body composition would be observed during the preseason, characterized by reductions in FM and the maintenance of or increases in FFM and LM. We further hypothesized that accumulated external and internal training load, as indicators of the overall training stimulus, would be associated with changes in body composition. We also expected that greater fatigue and less favorable wellness responses would be associated with smaller or less favorable body composition adaptations, reflecting differences in individual tolerance to and recovery from the accumulated training stimulus.

## 2. Materials and Methods

### 2.1. Study Design

A retrospective observational longitudinal study was conducted over a six-week preseason period to examine the relationship between accumulated training load (i.e., external and internal load), fatigue responses and changes in body composition in semi-professional football players. All data were retrospectively extracted from the team’s routine monitoring practices, which were implemented independently of the present research objectives. Players were monitored across 28 training sessions and seven matches during the 2025/26 preseason period, from 21 July to 31 August 2025. Training sessions were conducted between 9:00 a.m. and 10:30 a.m. under standardized conditions and were systematically monitored to quantify training load and fatigue-related variables. Body composition was assessed at three time points: during the first week of preseason, before players had completed any training sessions (Testing Session 1, TS1); during the fourth week of preseason (Testing Session 2, TS2); and at the beginning of the first week of the competitive season, corresponding to the seventh week of the study period (Testing Session 3, TS3). In addition, external load data were collected using GPS technology, while internal load and fatigue were assessed daily using questionnaires across all training sessions, resulting in a total of 661 individual observations. Body composition assessments were performed using BIA.

### 2.2. Participants

A total of 20 semi-professional football players (age: 26.9 ± 5.39 years; body mass: 74.06 ± 5.17 kg) participated in the study. The sample size was determined using convenience sampling based on the availability and accessibility of the participating team cohort. Therefore, no a priori sample size calculation was performed. All players belonged to the same team competing in the Spanish Fourth Division during the 2025/26 season. Goalkeepers were included in the analyses because their training load, fatigue, and body composition were monitored throughout the study period, as were the relationship among these variables. However, a complementary analysis excluding goalkeepers was performed and presented in Supplementary Table S1–S3, which show training load and fatigue-related variables for outfield players only. None of the participants sustained a long-term injury (>4 weeks) during the study, and all participants were injury-free at the beginning of the study. In addition, players were not involved in any other training programs aside from the training regimen imposed by the coach. The sample comprised five central midfielders, five wide midfielders, three central defenders, three full-backs, two forwards, and two goalkeepers. Player positions were reported to characterize the sample; however, position-specific analyses were not conducted due to the limited number of players within each positional subgroup, which would have reduced statistical power and increased the uncertainty of position-based comparisons. Only players who completed each monitored training session in full were included in the analyses. The study protocol was approved by the Ethics Committee of the Universidad de Extremadura (protocol code: 33/2024). All procedures were conducted in accordance with international ethical standards for research involving human participants. Data handling complied with the privacy, confidentiality, and data protection guidelines established by the American Psychological Association (2020).

### 2.3. External Load

Players’ external load during each training session was monitored using Polar Team Pro devices (Polar Electro Oy, Kempele, Finland). The system combines a 10 Hz GPS with a triaxial accelerometer and an integrated HR sensor. This technology has been developed to monitor physical activity in team sports and has previously demonstrated validity for measuring variables such as total distance covered (TD) and maximum running speed attained [39,40]. Moreover, it represents one of the most widely implemented systems for external load monitoring in football. The devices were placed on the players 10 min before to each training session and secured using a chest strap positioned at thoracic level. To reduce potential measurement variability, each player used the same GPS unit consistently throughout the study period. The external load variables considered for analysis were TD covered (in meters), high-speed running distance (HSR, 21–24 km·h^−1^), sprint distance (Sprint, >24 km·h^−1^), the number of accelerations greater than 3 m·s^−2^ (ACC), and the number of decelerations below −3 m·s^−2^ (DEC).

### 2.4. Rating of Perceived Exertion (RPE) and Session-RPE (sRPE)

The rating of perceived exertion (RPE) was assessed using a modified 10-point Borg scale, as previously proposed by Foster et al. [41]. Following each training session, players responded to the question, “How hard did you feel you worked during the session?”, providing a subjective assessment of their perceived exertion. The perceived exertion for each session was reported on a numerical scale ranging from 0 to 10. This procedure has been previously described and validated in football training contexts [42].

Session-RPE (sRPE) was also calculated by multiplying the RPE value by the total duration of each training session, expressed in minutes [43]. Session duration refers to the total length of the training session. Thus, a single arbitrary unit representing the magnitude of the global sRPE for each session is then calculated by the multiplying training intensity by the duration of training (min). For example, a session lasting 87 min with an RPE value of 4 would result in an sRPE of 348 arbitrary units (AU).

sRPE (A.U.)=RPE × session duration (min).

where sRPE is session*RPE; A.U. is Arbitrary Units; and RPE is Rating of Perceived Exertion.

### 2.5. Heart-Rate Exposure

The time spent by players in different HR zones was monitored as follows: time spent between 85% and 90% of maximum HR (HR85–90), time spent between 90% and 95% of maximum HR (HR90–95), and time spent between 95% and 100% of maximum HR (HR95–100). All HR zone variables were expressed in minutes. The HRmax values were updated daily to reflect any changes that occurred during the training sessions.

### 2.6. Fatigue

Fatigue-related variables were collected daily using a self-reported wellness questionnaire completed in the morning immediately after waking up and before the start of each training session. The questionnaire consisted of five items assessing fatigue, sleep quality, muscle soreness, stress, and mood. Each item was rated using a five-point Likert scale with one-point intervals, ranging from 1 to 5, where 1 indicated very poor and 5 indicated very good state [44]. This questionnaire was modified by adding an additional item assessing mental fatigue, defined as a psychobiological state characterized by feelings of tiredness and a lack of energy, resulting from prolonged periods of demanding cognitive activity. An additional item assessing mental fatigue was included in the questionnaire (“What is your level of mental fatigue related to the previous training session?”). Mental fatigue was defined as a state of tiredness resulting from increased cognitive demands [45]. This item was also rated using the same five-point Likert scale, ranging from 1 to 5. To ensure consistency with the overall wellness score, responses were coded so that higher scores reflected better perceived mental wellness and lower levels of mental fatigue. For clarity, player fatigue was analyzed in relation to the training load accumulated during the previous day’s training session.

### 2.7. Body Composition

Body composition assessments were conducted before training sessions, between 08:00 and 09:00 a.m. There was approximately a 22 to 24 h interval between the last training session and the body composition assessment performed after that session. Players reported that they had not taken any medication or pharmacological substance that could have affected the assessments during the 24 h prior to testing. All measurements were performed in a room with stable environmental conditions, and data collection procedures were conducted by the same researcher. Participants were instructed to undergo the test in their underwear, barefoot, and without any metal accessories. In addition, they were instructed to empty their bladders at least 30 min before the assessment and to refrain from eating or drinking for at least 2 h prior to the measurement. Although the assessment conditions were standardized, objective hydration status was not assessed, and fluid intake, and caffeine and alcohol consumption, were not systematically recorded.

Body composition was assessed using a multi-frequency, segmental BIA device (Tanita MC-780MA Segmental, Tanita Corporation, Barcelona, Spain). The device provides estimates of FM, FFM, LM, and SMM using the manufacturer’s built-in proprietary prediction algorithms. No additional or sport-specific prediction equation was applied by the researchers. Both absolute and relative values were recorded where applicable. Body composition was assessed at three time points during the preseason (TS1, TS2, and TS3).

### 2.8. Statistical Analysis

All analyses were conducted using RStudio (version 2024.12.0+467). The *dplyr* and *tidyr* packages were used for data management, *lme4* and *lmerTest* for linear mixed-effects models, *emmeans* for estimated marginal means and post-hoc contrasts, and *psych* for correlation analyses.

First, weekly changes in training-load and fatigue-related variables during preseason were examined using separate linear mixed-effects models (LMM). The random intercept accounted for the correlation among repeated observations from the same player. Random slopes were not included. Models were estimated using restricted maximum likelihood (REML), and degrees of freedom for fixed-effect tests were approximated using the Satterthwaite method. Thus, external load-related variables (TD, HSR, Sprint distance, Sprints, ACC, and DEC) and time spent in the HR zones were summed across sessions within each week. RPE, sRPE, and fatigue-related (Fatigue, sleep quality, muscle soreness, stress, mood and mental fatigue) indicators were averaged across the available sessions within each player-week. LMM were selected because they accommodate correlated repeated measurements and unequal numbers of observations across players [46]. Estimated marginal means (EMMs) and 95% confidence intervals (CIs) were calculated for each week, and consecutive and all-pairwise weekly comparisons were performed using *emmeans* with Tukey-adjusted *p* values. Percentage changes between consecutive weeks were also calculated.

Second, changes in body composition across the three testing sessions (TS1, TS2, and TS3) were also examined using separate LMM for FM, FFM, LM, and SMM. For each outcome, assessment occasion was included as a categorical fixed effect and player as a random intercept. Thus, the fixed effect estimated the mean differences among TS1, TS2, and TS3, whereas the player-specific random intercept accounted for repeated measurements within players. Models were estimated using REML, with Satterthwaite-adjusted degrees of freedom. EMMs and 95% CIs were obtained for each assessment occasion. All three pairwise contrasts (TS1 vs. TS2, TS1 vs. TS3, and TS2 vs. TS3) were examined, with Tukey-adjusted *p* values applied separately within each body-composition outcome. Differences between assessments were calculated with standardized effect sizes (ES) expressed as Cohen’s d and their 95% CIs [47]. Cohen’s d was calculated by dividing the estimated pairwise contrast by the residual standard deviation from the corresponding mixed-effects model. Effect-size magnitudes were interpreted according to Cohen’s conventional thresholds using the absolute value of d: negligible (<0.20), small (0.20–0.49), medium (0.50–0.79), and large (≥0.80).

Third, associations between training exposure and body composition from TS1 to TS3 were initially explored using Spearman correlations (*ρ)*. Spearman’s correlation was used to quantify the strength and direction of monotonic associations; it does not require the association to be linear. Training exposure was calculated using sessions occurring strictly after the TS1 assessment date and before the TS3 assessment date. Changes in FM, FFM, LM, and SMM were calculated as TS3 minus TS1. Correlation coefficients were reported with 95% CIs, unadjusted *p* values, and false-discovery-rate-adjusted *p* values. In addition, a correlation matrix was visualized through a heatmap (see Section 3.3), employing a gradient color scale ranging from blue (negative correlation) to red (positive correlation), with white indicating no correlation. The magnitudes of the correlations were interpreted based on the following thresholds: [0.0–0.1] = trivial; [0.1–0.3] = small; [0.3–0.5] = moderate; [0.5–0.7] = large; [0.7–0.9] = very large; [0.9, 1.0] = nearly perfect [48]. Confidence intervals of 95% were used for the correlation values (*r*).

Separate linear regression models were used to examine whether each training-load or fatigue-related variable was associated with body composition at the end of preseason independently of baseline body composition. A separate model was fitted for each predictor–outcome combination. Each model included the body composition value at TS3 as the dependent variable, the corresponding TS1 value as a covariate, and one training load or fatigue indicator as the predictor. Predictors were standardized before analysis; therefore, the unstandardized regression coefficient (B) represented the expected difference in the TS3 outcome associated with a one-standard-deviation increase in the predictor, after controlling for the baseline value. Fully standardized regression coefficients (β) were additionally calculated to facilitate comparisons across predictors and outcomes. For each regression model, *p* values were adjusted using the Benjamini–Hochberg procedure. Model assumptions were evaluated using residual-versus-fitted plots, quantile–quantile plots of residuals, and assessments of residual homoscedasticity. Potential collinearity between baseline body composition and the exposure predictor was examined using variance inflation factors. Cook’s distance and additional influence diagnostics were used to identify potentially influential observations. Also, potentially influential observations were examined in sensitivity checks but were not automatically removed.

No missing values were imputed. For the LMM analyses, all available outcome observations were retained, and players with incomplete repeated measurements could contribute data from the assessment occasions or weeks for which valid observations were available. LMM estimation therefore accommodated unbalanced numbers of repeated observations under the missing-at-random assumption. Observations missing the outcome or any predictor required for a particular model were excluded from that model.

Finally, given that the study retrospectively included a fixed cohort of 20 players from one team, no a priori sample size calculation was performed. A sensitivity analysis was therefore conducted to determine the minimum ES detectable with the available sample. For a linear regression model including two explanatory variables—the corresponding TS1 value and one exposure predictor—with one predictor being tested, a two-sided α of 0.05 and 80% power, the sample was sufficient to detect an incremental effect of approximately (f^2^ = 0.44), equivalent to a partial (R^2^) of approximately 0.31. For a bivariate correlation, the approximate minimum detectable coefficient was (ρ = 0.59). Thus, the study was primarily powered to detect large associations. These calculations were based on the nominal α level and did not incorporate the additional loss of power resulting from false-discovery-rate correction.

All tests were two-sided, with statistical significance set at *p* < 0.05. Interpretation emphasized the magnitude and precision of the estimated effects, their 95% CIs, and Benjamini–Hochberg-adjusted *p* values rather than statistical significance alone.

## 3. Results

### 3.1. Evolution of Training Load and Fatigue-Related Variables

**Figure 1:**
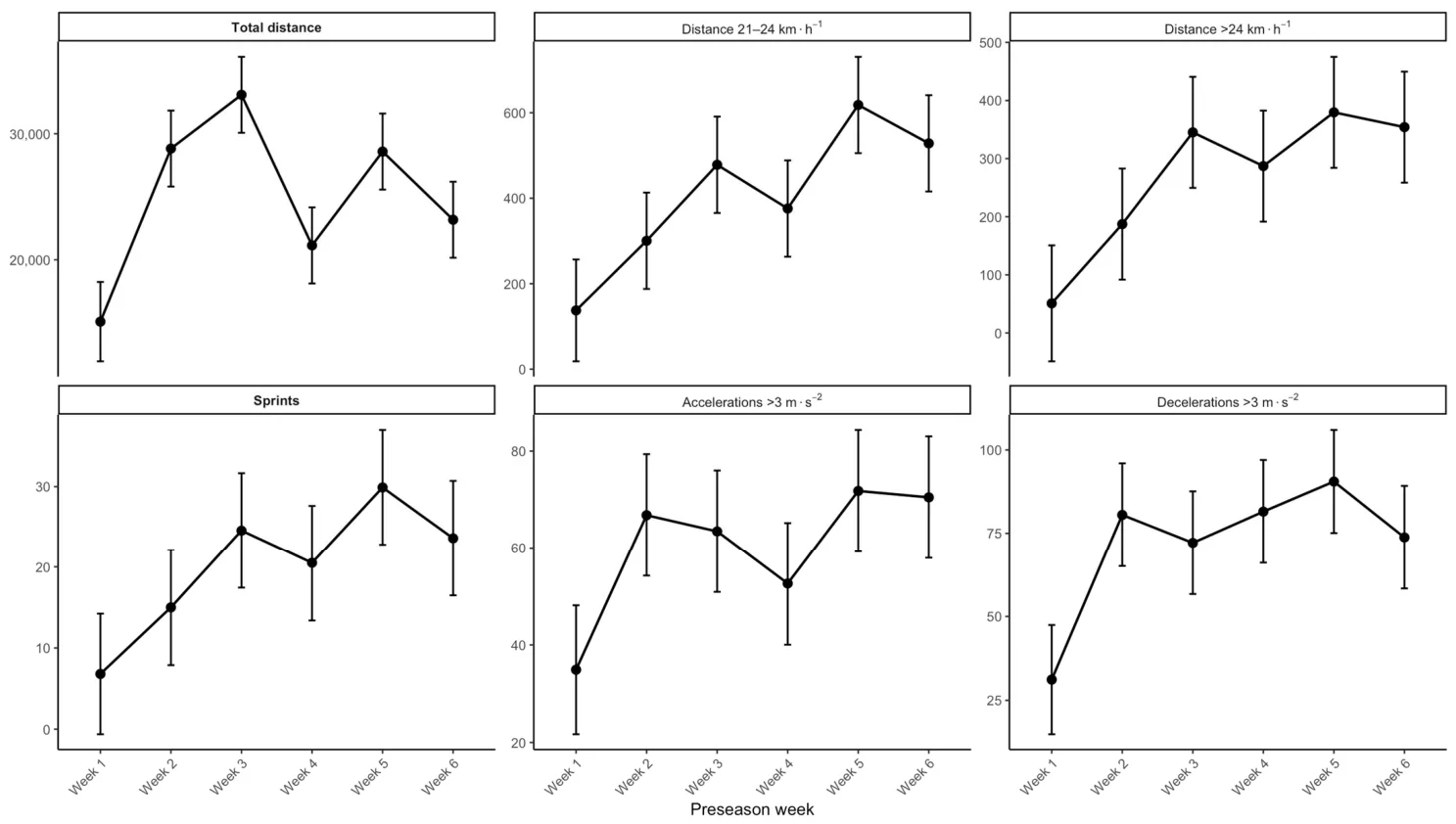
Evolution of external load variables during preseason. Note. High-speed running distance (HSR, 21–24 km·h^−1^); sprint distance (Sprint, >24 km·h^−1^); the number of accelerations greater than 3 m·s^−2^ (ACC); and the number of decelerations below −3 m·s^−2^ (DEC).

Figure 1 shows the evolution of external load-related variables across the preseason period. Overall, most variables showed a progressive increase throughout the preseason and followed a broadly similar temporal pattern, reaching their highest values in week 5. The only exception was TD, which peaked in week 3.

**Figure 2:**
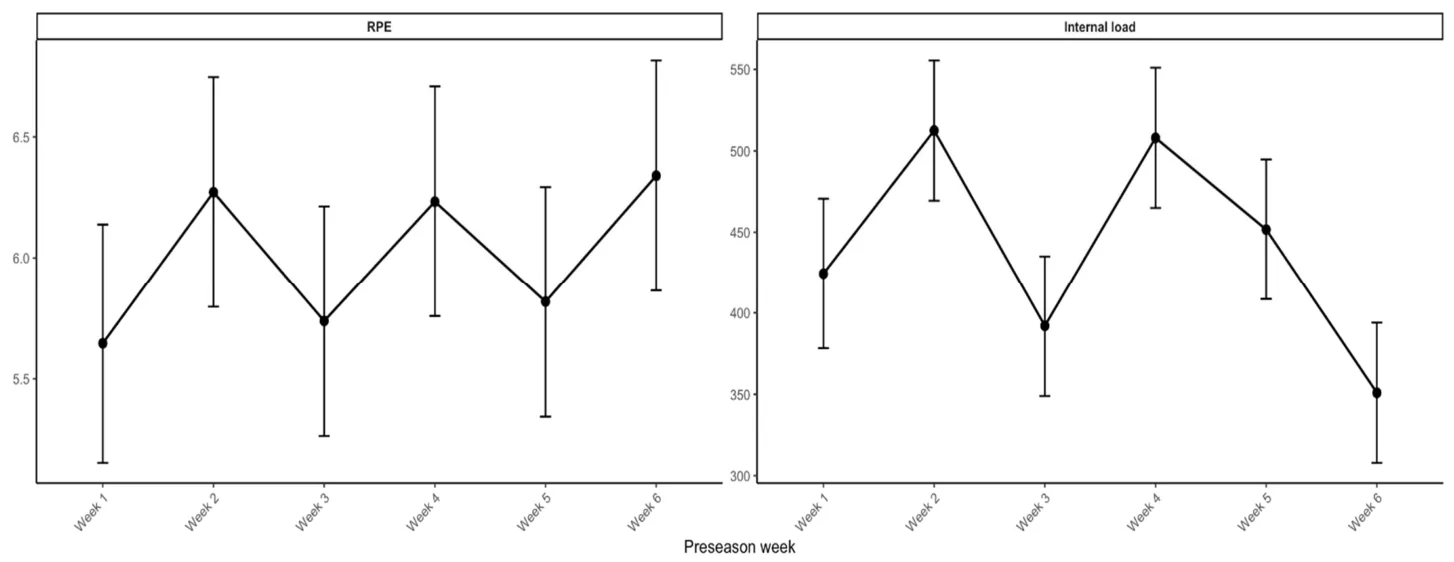
RPE and sRPE evolution during preseason. Note: RPE = Rate of Perceived Exertion.

Figure 2 shows the evolution of RPE and sRPE across the preseason. RPE presented its lowest values in week 1 and its highest values in week 6, whereas sRPE followed the opposite pattern, reaching its highest values in week 2 and its lowest values in week 6.

**Figure 3:**
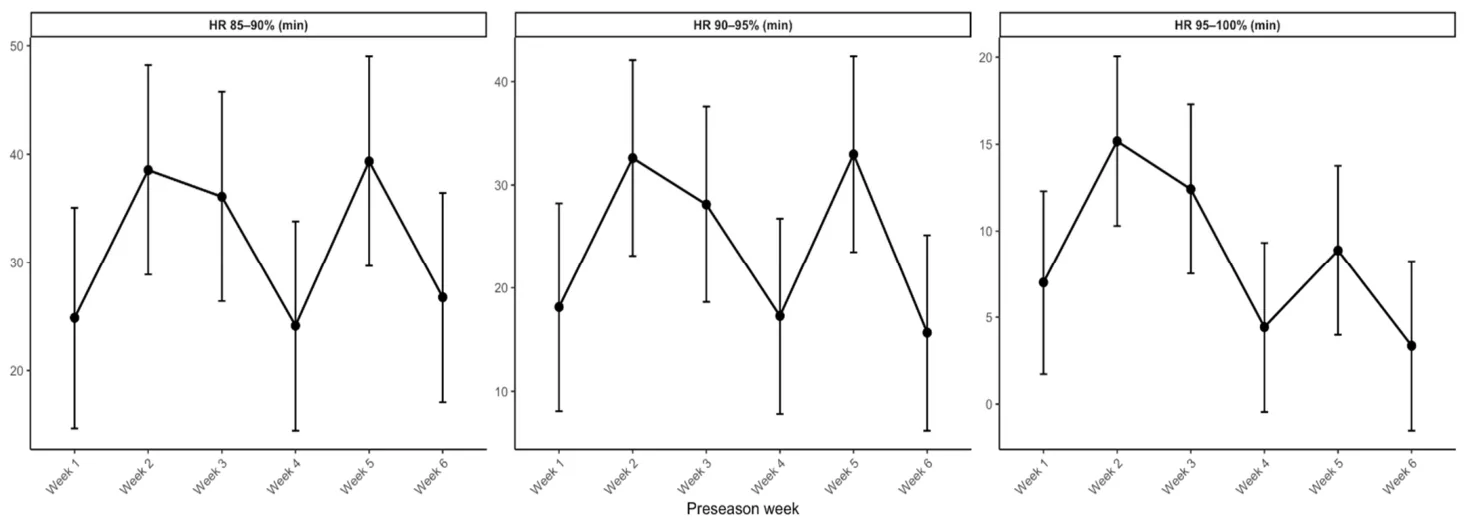
Evolution of time spent in HR zones during preseason. Note: HR = Heart Rate.

In addition, Figure 3 illustrates the evolution of time spent in the different HR zones. These variables followed a broadly similar temporal pattern, with the lowest values observed in weeks 4 and 6 and the highest values recorded in weeks 2 and 5.

**Figure 4:**
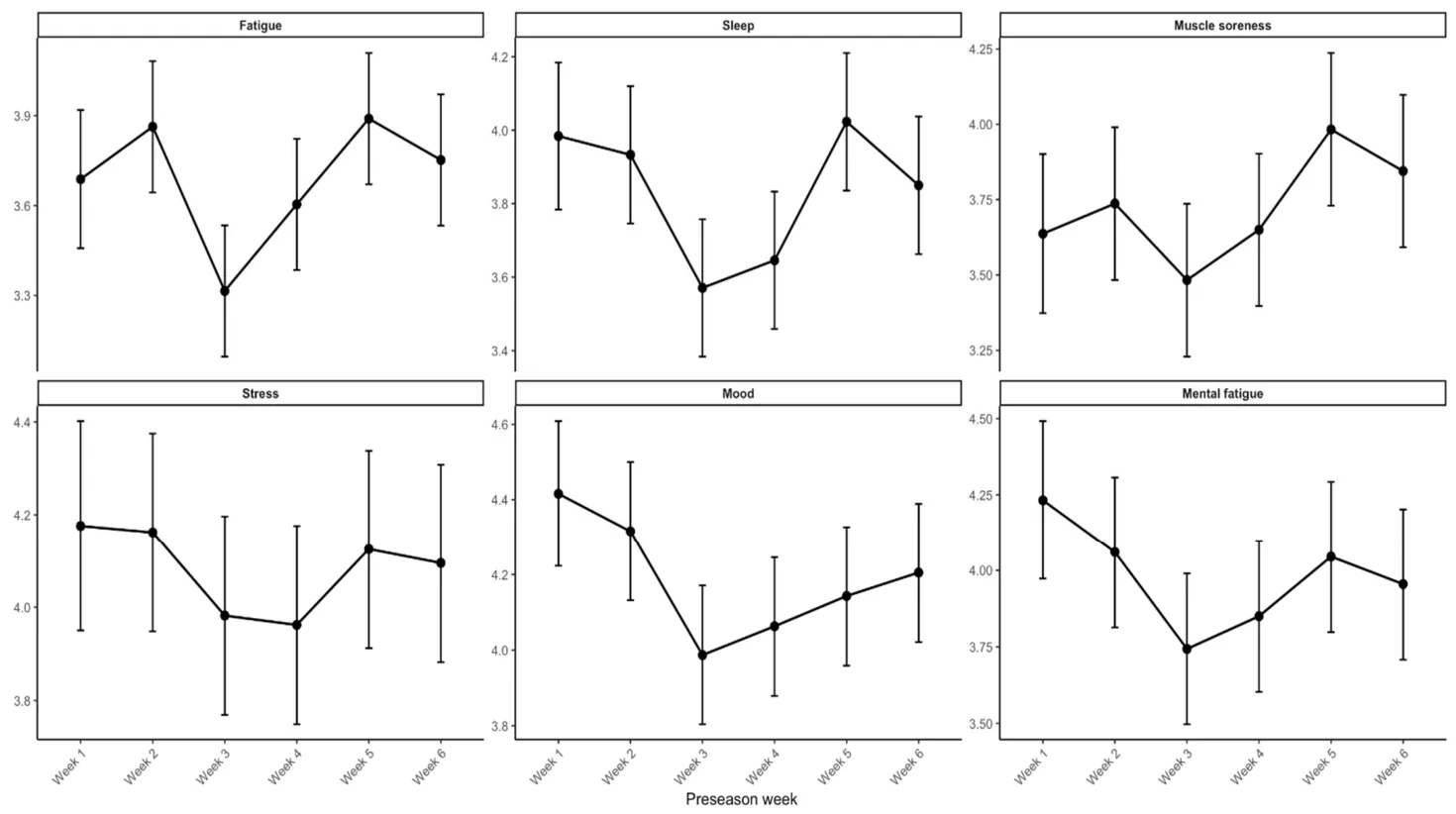
Evolution of wellness indicators during preseason.

Finally, Figure 4 shows the evolution of fatigue and wellness indicators across the preseason. These variables displayed a broadly similar temporal pattern, with fatigue, sleep quality, mood, and mental fatigue scores showing a decrease from the opening weeks and reaching their lowest recorded values in week 3.

**Figure 5:**
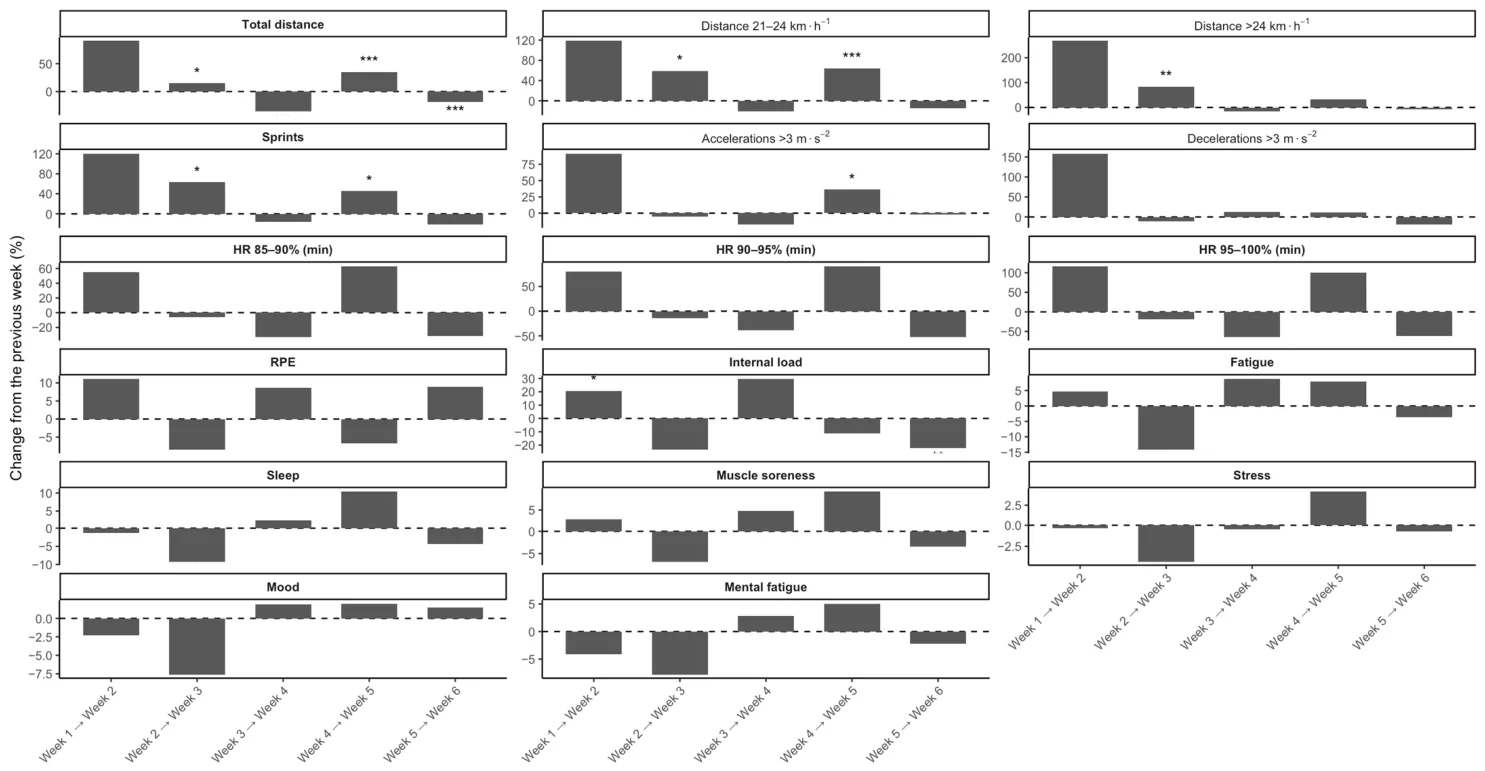
Weekly changes percentages in training load and fatigue-related variables during preseason. Note: HR = Heart Rate; RPE = Rate of Perceived Exertion; * *p* < 0.05; ** *p* < 0.01; *** *p* < 0.001; Tukey-adjusted comparisons. High-speed running distance (HSR, 21–24 km·h^−1^); sprint distance (Sprint, >24 km·h^−1^); the number of accelerations greater than 3 m·s^−2^ (ACC); and the number of decelerations below −3 m·s^−2^ (DEC).

Weekly percentage changes revealed a markedly fluctuating pattern in external load across the preseason (see Figure 5). Most external load indicators increased substantially from Week 1 to Week 2, followed by further significant increases from Week 2 to Week 3. External load generally declined from Week 3 to Week 4, before increasing again from Week 4 to Week 5. HR-based load indicators showed a comparable alternating pattern, although none of the consecutive-week changes reached statistical significance. sRPE increased from Week 1 to Week 2 and decreased from Week 5 to Week 6, whereas RPE remained comparatively stable. Fatigue and wellness indicators exhibited only modest week-to-week fluctuations, with declines in sleep, mood, and mental fatigue between Weeks 2 and 3 and partial recovery thereafter; however, no significant consecutive-week differences were observed.

### 3.2. Body Composition Evolution

Table 1 shows the evolution of body composition changes during the preseason. The results showed that the absolute and relative values of FM were significantly greater in TS3 than in TS2 (*p* < 0.001 both) and in TS1 (*p* < 0.001 both). In contrast, the absolute and relative values of FFM were significantly lower in TS3 than in TS2 (*p* < 0.001 both) and in TS1 (*p* < 0.01 and *p* < 0.001, respectively). The absolute and relative values of LM were also significantly lower in TS3 than in TS2 (*p* < 0.001 both) and in TS1 (*p* < 0.01 and *p* < 0.001, respectively). At TS2, SMM reached significantly greater values compared with TS1 (*p* < 0.01) and TS3 (*p* < 0.001).

### 3.3. Associations Between Training Load and Fatigue-Related Variables with Body Composition

**Figure 6:**
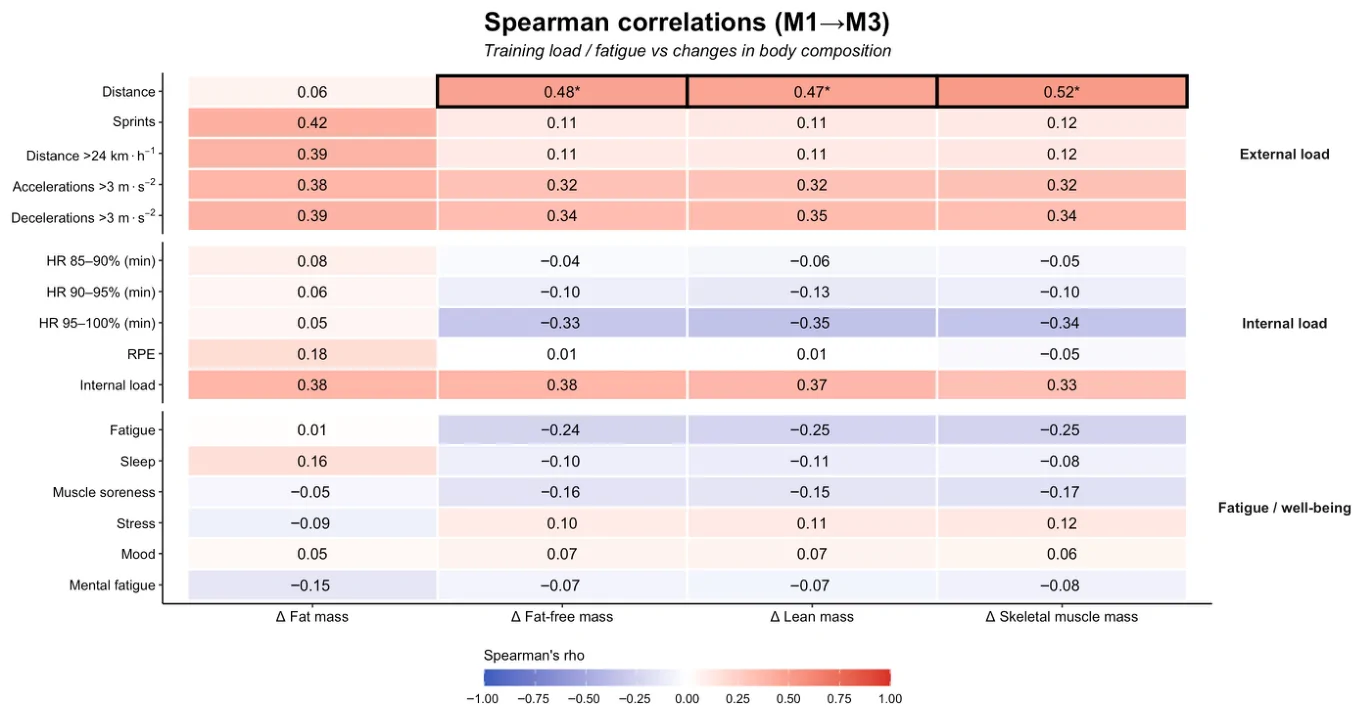
Heatmap of Spearman correlations training load and fatigue vs. change in body composition (TS1 → TS3). Note: RPE = Rate of Perceived Exertion. High-speed running distance (HSR, 21–24 km·h^−1^); sprint distance (Sprint, >24 km·h^−1^); the number of accelerations greater than 3 m·s^−2^ (ACC); and the number of decelerations below −3 m·s^−2^ (DEC). Cells show Spearman’s ρ for exploratory, unadjusted associations between accumulated training-load or fatigue-related variables and changes in body composition from TS1 to TS3. Asterisks indicate nominal, unadjusted *p* values; none of the associations remained statistically significant after Benjamini–Hochberg correction. These results should be considered hypothesis-generating; * *p* < 0.05.

In the exploratory unadjusted Spearman analyses (Figure 6), TD showed positive correlations with changes in FFM (*ρ* = 0.48, *p* < 0.05), LM (*ρ* = 0.47, *p* < 0.05), and SMM (*ρ* = 0.52, *p* < 0.05). Other external load indicators generally displayed small-to-moderate positive correlations with body composition changes, particularly ACC, DEC, Sprint distance, and sRPE, although these associations did not reach statistical significance. Time spent at 95–100% of maximal HR and RPE tended to be negatively associated with changes in lean tissue compartments, whereas the remaining fatigue and wellness indicators showed predominantly weak relationships.

Table 2 shows the associations between external training load indicators and body composition variables. None of the external load indicators were significantly associated with FM, FFM, LM, or SMM at TS3, and no association remained significant after the Benjamini–Hochberg correction. DEC showed the strongest positive association with LM together with a similar, albeit non-significant, tendency for FFM and SMM. TD, which was positively correlated with changes in the lean tissue outcomes in the unadjusted Spearman analyses, showed substantially weaker associations after controlling for baseline body composition.

Table 3 shows the associations between internal training load indicators and body composition variables. These indicators were likewise not significantly associated with any body composition outcome after baseline adjustment. Accumulated sRPE showed the clearest positive tendencies for FFM and LM. However, these estimates were imprecise and did not reach statistical significance (*p* ≈ 0.12–0.13). Time spent within the 85–90% and 90–95% HR zones were also largely unrelated to body composition, whereas exposure to the 95–100% zone tended to be negatively associated with FFM, LM, and SMM.

Table 4 shows the associations between fatigue indicators and body composition. Overall, fatigue and wellness indicators showed weak associations with body composition at TS3. Perceived fatigue exhibited the most consistent negative pattern across the lean-tissue outcomes. Sleep, muscle soreness, stress, mood, and mental fatigue showed predominantly small and inconsistent associations with all four body composition variables. Collectively, the fatigue and wellness measures contributed very little additional explanatory variance after accounting for baseline body composition, and none of the results supported an independent association with preseason body composition outcomes.

### 3.4. Sensitivity Analysis

Sensitivity analyses excluding the two goalkeepers produced body-composition changes and weekly temporal patterns that were broadly consistent with those obtained for the complete cohort. From TS1 to TS3, outfield players showed an increase of 1.19 kg in FM and decreases of 0.81 kg in FFM, 0.78 kg in LM, and 0.47 kg in SMM. The shapes of the weekly trajectories were highly similar between the complete and outfield-only cohorts, although the absolute values of several high-intensity external-load indicators increased after goalkeeper exclusion.

In the outfield-only Spearman analyses, TD was positively correlated with changes in FFM (ρ = 0.48, *p* = 0.045) and SMM (ρ = 0.52, *p* = 0.027), while time accumulated at 95–100% of maximal HR was negatively correlated with changes in LM (ρ = −0.49, *p* = 0.038) and SMM (ρ = −0.48, *p* = 0.044). None of these exploratory associations remained statistically significant after Benjamini–Hochberg correction (all adjusted *p*-values ≥ 0.58).

None of the 64 baseline-adjusted regression models was statistically significant in the outfield-only sample, either before or after multiple-comparison correction. Although some individual coefficients changed after goalkeeper exclusion, the overall inferential conclusion was unchanged. Complete results are provided in Supplementary Tables S1–S3.

## 4. Discussion

The aims of the present study were (i) to compare the evolution of external load, internal load, HR exposure, fatigue-related variables, and body composition, assessed using BIA, throughout the football preseason period; and (ii) to examine the associations between external and internal training load, fatigue-related variables, and changes in body composition throughout the preseason. The main findings were that FM increased, whereas FFM, LM, and SMM decreased by the end of preseason, despite transient improvements at the intermediate assessment. More importantly for the second study aim, the baseline-adjusted and multiplicity-corrected analyses did not provide statistically robust evidence that accumulated external load, internal load, or subjective fatigue-related variables were independently associated with body composition at TS3. Although some preliminary unadjusted correlations were observed, these did not remain significant after the more rigorous adjusted analyses and were therefore interpreted as hypothesis-generating.

### 4.1. Evolution of Training Load and Fatigue-Related Variables

The preseason is characterized by high and progressively organized training demands aimed at restoring physical fitness before competition. However, previous research has shown that the temporal evolution of training load may vary depending on the variables used for quantification [49]. Some studies have reported progressive increases in internal load, monotony, and strain during the initial weeks of preseason, followed by a tapering period before competition [50], whereas others have observed relatively stable weekly external load despite considerable day-to-day variation [51]. Similarly, Azcárate et al. [52] showed that preseason is associated with greater perceived respiratory and muscular load than the competitive season, with muscular load exhibiting the largest fluctuations.

In the present study, fatigue-related variables showed a broadly similar temporal pattern, reaching their lowest values in week 3. Although fewer studies have examined the evolution of fatigue during preseason in football, previous evidence suggests that this period imposes greater overall demands on players than the in-season period [53]. These findings reinforce the notion that preseason training should be interpreted as a dynamic process rather than a homogeneous loading period. Differences in periodization strategies, match exposure, recovery scheduling, and individual responses may explain the variability observed among studies and further support the need for comprehensive athlete monitoring throughout the preseason.

### 4.2. Evolution of Body Composition and Training Load

The present study revealed changes in body composition throughout the preseason, characterized by increased FM and reductions in FFM, LM, and SMM after the six-week training period. Although this pattern contrasts with the predominant findings reported in elite football, where preseason training is generally associated with reductions in FM and maintenance or increases in lean tissue [20,27,54], the available evidence is not entirely consistent.

Several longitudinal studies have shown that the greatest changes in body composition occur between the beginning of preseason and the start of competition, with subsequent stabilization or partial regression throughout the competitive season [54,55,56]. In football, these adaptations frequently occur without substantial changes in total body mass, reflecting shifts in body composition rather than overall weight [20]. However, previous research has also demonstrated that favorable adaptations are not universal. Clemente et al. [29] reported a slight increase in FM accompanied by a reduction in LM following a 10-week preseason in professional football players, while Xiong [57] found no significant changes in anthropometric characteristics despite improvements in physical performance. Similarly, Staśkiewicz-Bartecka et al. [56] observed that trunk fat progressively increased across the competitive macrocycle, with body composition responses varying according to the phase of the season and playing position.

Collectively, these findings suggest that changes in body composition during the preseason may be highly context dependent. In addition to the external training load monitored by GPS, body composition may be associated with multiple interacting factors, including dietary intake, energy availability, recovery status, resistance-training characteristics, competitive level, age, playing position, and individual biological variability that may influence changes in body composition [29,54]. Importantly, GPS-derived variables primarily quantify locomotor and external activity and therefore represent only one component of the overall training stimulus. Furthermore, the present study did not assess dietary intake or energy availability, both of which are recognized as key determinants of body composition adaptations during periods of intensified training [58,59]. Consequently, the observed changes cannot be attributed solely to the monitored training stimulus. Furthermore, the inclusion of an intermediate assessment (TS2) revealed that body composition adaptations were not linear, with initial improvements followed by deterioration before the competitive season. This finding supports current recommendations advocating longitudinal athlete monitoring, as repeated assessments may identify transient physiological adaptations that would remain undetected using only pre- and post-preseason evaluations [9].

The observed changes in body composition should be interpreted cautiously considering the physiological and methodological characteristics of BIA. In particular, the simultaneous increase in FM and reductions in FFM, LM, and SMM over a relatively short preseason period may reflect genuine changes in body composition but may also be influenced by variations in hydration status and glycogen stores. Changes in total body water can affect the electrical conductivity measured by BIA and consequently influence the estimation of fat-free compartments [34,60].

### 4.3. Relationship Between Training Load, Fatigue and Body Composition

The primary analyses did not provide statistically robust evidence that accumulated external load, internal load, or subjective fatigue-related variables were independently associated with body composition at the end of preseason. After controlling for the corresponding baseline body composition value, none of the associations remained statistically significant after Benjamini–Hochberg correction. Therefore, the adjusted and multiplicity-corrected analyses do not support an independent association between any individual monitored exposure and FM, FFM, LM, or SMM in the present sample. Although training load is widely recognized as an important component of the training stimulus, the available evidence suggests that no single load metric consistently accounts for differences in body composition responses. Previous investigations have reported associations between preseason energy expenditure and changes in FM and FFM [20], while Clemente et al. [29] observed that accumulated sprint distance was more strongly associated with body composition adaptations than total running volume. Together, these findings indicate that the relationship between training load and body composition is likely dependent on the specific training stimulus being considered.

The exploratory Spearman correlation analyses revealed several moderate associations between accumulated external load and changes in lean tissue. Specifically, TD was moderately and positively correlated with changes in FFM (*ρ* = 0.48, *p* < 0.05), LM (*ρ* = 0.47, *p* < 0.05), and SMM (*ρ* = 0.52, *p* < 0.05). Our findings indicate that greater overall running volumes were moderately associated with more favorable changes in lean tissue in the unadjusted analyses. However, these associations should be interpreted cautiously because they were obtained from unadjusted analyses and did not remain statistically significant after accounting for baseline body composition and multiple comparisons. Indeed, in the adjusted regression models, the association between TD and FFM was substantially attenuated (B = 0.33 kg, 95% CI: −0.20 to 0.86, *p* = 0.207), as were the associations with LM (B = 0.30 kg, 95% CI: −0.21 to 0.81, *p* = 0.231) and SMM (B = 0.18 kg, 95% CI: −0.12 to 0.48, *p* = 0.218). This discrepancy between the unadjusted correlations and adjusted models suggests that the initial relationships may have been partly explained by differences in baseline body composition rather than representing an independent association with accumulated running exposure.

Previous studies have reported heterogeneous findings regarding the relationship between training exposure and body composition. Some investigations have observed associations between preseason energy expenditure or specific high-intensity load measures and changes in FM or FFM [20,29]. Conversely, Varley et al. [27] reported improvements in body composition without meaningful associations between GPS-derived load variables and changes in lean or FM. These differences may reflect variation in study populations, training programs, analytical approaches, dietary intake, resistance-training exposure, and the duration and timing of body composition assessments.

Fatigue-related variables (assessed using the wellness questionnaire) also showed predominantly weak associations with body composition. Perceived fatigue exhibited the most consistent negative pattern across the lean-tissue outcomes, with estimated associations of −0.38 kg for FFM (95% CI: −0.88 to 0.12, *p* = 0.131), −0.36 kg for LM (95% CI: −0.84 to 0.13, *p* = 0.142), and −0.22 kg for SMM (95% CI: −0.50 to 0.06, *p* = 0.119) per 1-SD increase in fatigue. A similar negative tendency was observed for LM, indicating that greater accumulated fatigue was associated with slightly less favorable changes in lean tissue in the present sample. Different physiological adaptations may also respond to different components of the training stimulus. Internal-load measures such as sRPE and TRIMP have been associated more closely with aerobic fitness adaptations, whereas HSR and sprint exposure may be more relevant to neuromuscular performance [61,62]. GPS-derived variables predominantly quantify locomotor activity and may not capture resistance-training stimuli, dietary energy balance, macronutrient intake, hydration, or recovery-related factors that can influence body composition. Consequently, accumulated GPS or subjective load measures may represent only part of the adaptive environment relevant to changes in FM and lean tissue.

These findings suggest that expecting a linear relationship between a single training load metric and body composition may oversimplify the complex nature of physiological adaptation. Internal and external load variables quantify different aspects of the training stimulus and should therefore be considered complementary rather than interchangeable [9]. Moreover, the physiological demands of football vary according to the type of training task, playing position, and microcycle structure. For example, sRPE has shown stronger associations with internal physiological stress during small-sided games and conditioning drills than during tactical training [63], while external load profiles differ substantially according to playing position and match-day proximity, particularly for HSR and sprint-related metrics [64,65]. Consequently, the accumulated load quantified over an entire preseason may not adequately capture the specific stimuli associated with changes in body composition, reinforcing the need for multidimensional athlete monitoring.

The lack of statistically robust associations should not be interpreted as evidence that training exposure is unrelated to body composition under all conditions. The relatively small sample, uncertainty in the effect estimates, large number of exposure–outcome combinations, and absence of several potentially important covariates limited the strength of the conclusions. Rather, the present results indicate that none of the monitored variables demonstrated an independent association with body composition that was sufficiently precise and robust to withstand baseline adjustment and multiple-comparison correction. The preliminary unadjusted correlations should therefore be considered hypothesis-generating and require confirmation in larger, prospectively designed studies incorporating nutritional intake, energy availability, hydration status, and resistance-training exposure.

### 4.4. Limitations and Future Directions

Some limitations should be acknowledged when interpreting the present findings: (i) Body composition was assessed using BIA. Although this method is practical and has demonstrated acceptable validity when standardized protocols are followed, it remains less accurate than criterion techniques such as DXA, particularly for detecting small longitudinal changes [60,66]. (ii) Several factors known to influence body composition were not monitored throughout the preseason. Dietary intake, energy availability, hydration status, sleep quantity and quality, caffeine and alcohol consumption, and resistance training characteristics may have contributed to the observed changes and could partially explain the lack of associations between training load and body composition. Therefore, variations in hydration status and other pre-assessment conditions may have contributed to the observed changes in BIA. Future research should include dietary assessment and measures of energy availability when examining preseason changes in body composition. (iii) A new item (assessing mental fatigue) was added to the wellness questionnaire, but it had not been validated. In future studies, it would be interesting to validate this item in order to provide the scientific community with new empirical tools. (iv) The relatively small sample size (*n* = 20) is a major limitation of this study. Since the sample size was determined based on the availability of the participating team’s cohort, rather than through an a priori sample size calculation, the study may have had limited statistical power to detect weak-to-moderate associations, especially given the number of variables analyzed. (v) Playing position represents a potential source of heterogeneity in external training load that was not explicitly modeled in the main statistical analyses. The primary analyses included the two goalkeepers because the target cohort comprised the complete monitored squad. However, goalkeepers have substantially different locomotor demands compared with outfield players, and their inclusion affected the magnitude of some GPS-derived exposure estimates. A complementary outfield-only sensitivity analysis was therefore performed, which produced the same overall inferential conclusion, with no baseline-adjusted association remaining statistically significant after correction for multiple comparisons. Nevertheless, some individual coefficients changed in magnitude or direction, indicating that position-related heterogeneity cannot be disregarded. The small number of players within each positional subgroup prevented reliable position-specific modelling or the estimation of position-by-time interactions. (vi) Finally, the absence of a control or comparison group is another limitation to consider. All participants belonged to the same team and followed the same preseason training program. Therefore, the study design does not allow the specific effects of the monitored training stimulus to be distinguished from other time-dependent or contextual factors that may have influenced body composition.

### 4.5. Practical Applications

The present findings indicate that changes in body composition observed during the preseason were not significantly associated with the monitored external load, internal load, or fatigue-related variables. Therefore, these measures alone may not fully characterize the factors associated with individual changes in body composition. From a practical perspective, body composition may benefit from being monitored regularly throughout the preseason alongside training-load and fatigue-related measures to identify individual changes over time.

Based on the multifactorial nature of body composition and previous evidence, practitioners may also consider complementary information regarding nutritional intake, energy availability, resistance-training exposure, hydration, and recovery when interpreting changes in body composition. However, these factors were not directly assessed in the present study and therefore should be considered as broader recommendations rather than conclusions directly supported by our data. An integrated monitoring approach may provide a more comprehensive framework for interpreting individual body-composition changes during the preseason.

## 5. Conclusions

In conclusion, significant changes in body composition were observed over the preseason period in semi-professional football players, characterized by increased FM and reduced FFM, LM, and SMM by the beginning of the competitive season. However, the baseline-adjusted and multiplicity-corrected analyses provided no statistically robust evidence that accumulated external load, internal load, or subjective fatigue-related variables were independently associated with body composition at the end of preseason. Preliminary unadjusted correlations should be regarded as hypothesis-generating and require confirmation in larger studies. Given the observational design, limited sample size, and absence of information on dietary intake, energy availability, hydration, and resistance-training exposure, the observed body composition changes cannot be attributed solely to the monitored training stimulus.

Therefore, training load monitoring may be complemented by consideration of nutritional intake, energy availability, hydration, and recovery-related factors when interpreting changes in body composition during the preseason. Future studies including dietary intake and energy availability are warranted to clarify the mechanisms underlying these adaptations.

## Figures and Tables

**Table 1:** Evolution of body composition variables during preseason.

	TS1		TS2		TS3		*p*	*d* (CI95%)		
	Coeff (SE)	CI95%	Coeff (SE)	CI95%	Coeff (SE)	CI95%		TS1 vs. TS2	TS1 vs. TS3	TS2 vs. TS3
**FM (kg)**	5.93 (0.43)	(5.04, 6.83)	5.45 (0.43)	(4.56, 6.34)	7.04 (0.43)	(6.15, 7.93)	a *, b ***, c ***	0.81 (0.14, 1.49)	−1.85 (−2.61, −1.10)	−2.67 (−3.51, −1.82)
**FM (%)**	8.02 (0.59)	(6.8, 9.24)	7.34 (0.59)	(6.12, 8.56)	9.51 (0.59)	(8.29, 10.73)	a *, b ***, c ***	0.86 (0.18, 1.54)	−1.89 (−2.65, −1.13)	−2.75 (−3.61, −1.90)
**FFM (kg)**	68.12 (1.15)	(65.72, 70.53)	68.95 (1.15)	(66.55, 71.36)	67.26 (1.15)	(64.86, 69.67)	a **, b **, c ***	−1.08 (−1.78, −0.38)	1.12 (0.42, 1.81)	2.19 (1.40, 2.99)
**FFM (%)**	91.99 (0.59)	(90.76, 93.21)	92.66 (0.59)	(91.44, 93.88)	90.49 (0.59)	(89.27, 91.71)	a *, b ***, c ***	−0.86 (−1.54, −0.18)	1.90 (1.15, 2.66)	2.76 (1.91, 3.62)
**LM (kg)**	64.75 (1.1)	(62.46, 67.05)	65.53 (1.1)	(63.24, 67.83)	63.93 (1.1)	(61.64, 66.23)	a **, b **, c ***	−1.05 (−1.75, −0.36)	1.11 (0.41, 1.80)	2.16 (1.37, 2.95)
**LM (%)**	87.43 (0.56)	(86.26, 88.6)	88.07 (0.56)	(86.9, 89.24)	86.00 (0.56)	(84.83, 87.17)	a *, b ***, c ***	−0.85 (−1.53, −0.17)	1.90 (1.14, 2.65)	2.75 (1.89, 3.60)
**SMM (kg)**	38.58 (0.65)	(37.22, 39.94)	39.04 (0.65)	(37.68, 40.4)	38.08 (0.65)	(36.72, 39.45)	a **, b **, c ***	−1.06 (−1.75, −0.37)	1.14 (0.44, 1.84)	2.20 (1.40, 2.99)

Note. Coeff = Coefficient; SE = Standard error; CI = Confidence intervals; TS = Testing session; FM Fat mass; FFM = Fat-free mass; LM = Lean mass; SMM = Skeletal muscle mass; kg = kilograms; a = significant differences between TS1 and TS2; b = significant differences between TS1 and TS3; c = significant differences between TS2 and TS3; * *p* < 0.05; ** *p* < 0.01; *** *p* < 0.001.

**Table 2:** Associations of accumulated external training load indicators with body composition variables.

	B (95% CI)	β	*p*	qBH	R^2^	Adjusted R^2^
*Fat mass*						
TD	0.18 (−0.25, 0.61)	0.090	0.392	0.808	0.823	0.802
Sprint	0.31 (−0.11, 0.72)	0.55	0.138	0.659	0.838	0.819
Sprints	0.32 (−0.09, 0.73)	0.161	0.122	0.659	0.840	0.821
ACC	0.25 (−0.20, 0.69)	0.125	0.259	0.659	0.829	0.809
DEC	0.32 (−0.09, 0.74)	0.163	0.119	0.659	0.840	0.822
*Fat-free mass*						
TD	0.33 (−0.20, 0.86)	0.063	0.207	0.659	0.964	0.960
Sprint	0.17 (−0.39, 0.73)	0.032	0.532	0.832	0.961	0.957
Sprints	0.19 (−0.37, 0.75)	0.036	0.486	0.832	0.961	0.957
ACC	0.30 (−0.25, 0.86)	0.058	0.265	0.659	0.963	0.959
DEC	0.42 (−0.13, 0.96)	0.080	0.123	0.659	0.966	0.962
*Lean mass*						
TD	0.30 (−0.21, 0.81)	0.060	0.231	0.659	0.963	0.958
Sprint	0.17 (−0.37, 0.71)	0.033	0.522	0.832	0.960	0.956
Sprints	0.19 (−0.35, 0.72)	0.037	0.478	0.832	0.961	0.956
ACC	0.29 (−0.25, 0.82)	0.058	0.269	0.659	0.962	0.958
DEC	0.41 (−0.11, 0.94)	0.082	0.115	0.659	0.965	0.961
*Skeletal muscle mass*						
TD	0.18 (−0.12, 0.48)	0.061	0.218	0.659	0.964	0.960
Sprint	0.09 (−0.23, 0.40)	0.030	0.559	0.832	0.961	0.960
Sprints	0.10 (−0.22, 0.42)	0.033	0.516	0.832	0.962	0.957
ACC	0.15 (−0.17, 0.46)	0.049	0.344	0.734	0.963	0.959
DEC	0.21 (−0.10, 0.52)	0.070	0.174	0.659	0.964	0.961

Note. B = Non-standardized coefficient B; β = Standardized β coefficient; qBH = q-value adjusted using Benjamini–Hochberg; TD = Total distance; Sprint = Sprint running distance; Sprints = numbers of sprints; ACC = Accelerations; DEC = Decelerations.

**Table 3:** Associations of accumulated internal training load indicators with body composition variables.

	B (95% CI)	β	*p*	qBH	R^2^	Adjusted R^2^
*Fat mass*						
RPE	0.24 (−0.19, 0.66)	0.119	0.260	0.659	0.829	0.809
Session*RPE	0.28 (−0.15, 0.71)	0.140	0.191	0.659	0.833	0.814
HR 85–90%	0.11 (−0.34, 0.56)	0.056	0.608	0.831	0.818	0.797
HR 90–95%	0.13 (−0.32, 0.58)	0.065	0.549	0.831	0.819	0.798
HR 95–100%	0.06 (−0.39, 0.50)	0.029	0.789	0.890	0.816	0.794
*Fat-free mass*						
RPE	0.05 (−0.57, 0.66)	0.009	0.878	0.915	0.963	0.956
Session*RPE	0.40 (−0.12, 0.93)	0.078	0.121	0.659	0.966	0.959
HR 85–90%	−0.04 (−0.58, 0.50)	−0.007	0.882	0.915	0.960	0.956
HR 90–95%	−0.10 (−0.63, 0.44)	−0.018	0.711	0.890	0.961	0.956
HR 95–100%	−0.32 (−0.83, 0.19)	−0.061	0.205	0.659	0.964	0.960
*Lean mass*						
RPE	0.04 (−0.55, 0.63)	0.008	0.886	0.915	0.959	0.955
Session*RPE	0.38 (−0.13, 0.89)	0.076	0.133	0.659	0.965	0.960
HR 85–90%	−0.06 (−0.58, 0.46)	−0.011	0.820	0.905	0.960	0.955
HR 90–95%	−0.11 (−0.63, 0.40)	−0.023	0.649	0.866	0.960	0.955
HR 95–100%	−0.33 (−0.82, 0.16)	−0.066	0.173	0.659	0.964	0.959
*Skeletal muscle mass*						
RPE	−0.01 (−0.27, 0.35)	0.014	0.782	0.890	0.960	0.956
Session*RPE	0.19 (−0.12, 0.49)	0.062	0.213	0.659	0.964	0.960
HR 85–90%	−0.02 (−0.33, 0.28)	−0.008	0.875	0.915	0.96	0.956
HR 90–95%	−0.04 (−0.35, 0.26)	−0.015	0.762	0.890	0.961	0.956
HR 95–100%	−0.18 (−0.46, 0.11)	−0.060	0.212	0.659	0.964	0.960

Note: B = Non-standardized coefficient B; β = Standardized β coefficient; qBH = q-value adjusted using Benjamini–Hochberg; RPE = Ratio of perceived exertion; HR = Heart rate.

**Table 4:** Associations of accumulated fatigue indicators with body composition variables.

	B (95% CI)	β	*p*	qBH	R^2^	Adjusted R^2^
*Fat mass*						
Fatigue	0.11 (−0.32, 0.55)	0.057	0.592	0.832	0.818	0.797
Sleep	0.13 (−0.30, 0.56)	0.066	0.532	0.831	0.819	0.798
Muscle Soreness	−0.08 (−0.53, 0.37)	−0.040	0.711	0.890	0.817	0.795
Stress	0.02 (−0.42, 0.47)	0.012	0.912	0.926	0.815	0.793
Mood	−0.09 (−0.52, 0.35)	−0.044	0.682	0.890	0.817	0.795
Mental fatigue	−0.06 (−0.50, 0.38)	−0.028	0.793	0.890	0.816	0.794
*Fat-free mass*						
Fatigue	−0.38 (−0.88, 0.12)	−0.072	0.131	0.659	0.965	0.961
Sleep	−0.14 (−0.67, 0.39)	−0.027	0.587	0.832	0.960	0.956
Muscle Soreness	−0.29 (−0.83, 0.24)	−0.06	0.265	0.659	0.963	0.959
Stress	0.17 (−0.36, 0.70)	0.03	0.507	0.832	0.961	0.957
Mood	0.07 (−0.47, 0.62)	0.014	0.780	0.890	0.960	0.956
Mental fatigue	−0.26 (−0.78, 0.26)	−0.049	0.310	0.700	0.963	0.958
*Lean mass*						
Fatigue	−0.36 (−0.84, 0.13)	−0.071	0.142	0.659	0.960	0.955
Sleep	−0.13 (−0.64, 0.38)	−0.025	0.607	0.832	0.960	0.956
Muscle Soreness	−0.28 (−0.79, 0.24)	−0.055	0.274	0.659	0.962	0.958
Stress	0.17 (−0.34, 0.68)	0.035	0.483	0.832	0.961	0.956
Mood	0.08 (−0.45, 0.60)	0.015	0.763	0.890	0.960	0.956
Mental fatigue	−0.24 (−0.75, 0.26)	−0.049	0.317	0.700	0.962	0.957
*Skeletal muscle mass*						
Fatigue	−0.22 (−0.50, 0.06)	−0.074	0.119	0.659	0.966	0.962
Sleep	−0.07 (−0.37, 0.23)	−0.025	0.611	0.832	0.961	0.957
Muscle Soreness	−0.18 (−0.48, 0.13)	−0.059	0.235	0.659	0.964	0.960
Stress	0.10 (−0.20, 0.40)	0.034	0.487	0.832	0.962	0.957
Mood	0.04 (−0.27, 0.35)	0.014	0.782	0.890	0.960	0.956
Mental fatigue	−0.15 (−0.45, 0.14)	−0.052	0.278	0.659	0.963	0.959

Note: B = Non-standardized coefficient B; β = Standardized β coefficient; qBH = q-value adjusted using Benjamini–Hochberg.

## Data Availability

The raw data supporting the conclusions of this article will be made available by the authors on request.

## Supplementary Materials

The following supporting information can be downloaded at https://www.mdpi.com/article/10.3390/jfmk11040410/s1, Table S1: Weekly evolution of external load-related variables during preseason after excluding goalkeepers; Table S2: Weekly evolution of internal load-related variables and heart rate during preseason after excluding goalkeepers, Table S3: Weekly evolution of fatigue-related variables during preseason after excluding goalkeepers.

## Abbreviations

The following abbreviations are used in this manuscript:

ACC	Accelerations
BIA	Bioelectrical Impedance Analysis
DEC	Decelerations
DXA	Dual-energy X-ray Absorptiometry
ES	Effect size
FFM	Fat- free mass
FM	Fat mass
GPS	Global Positioning System
HR	Heart rate
HSR	High-speed running
LM	Lean mass
LMM	Linear mixed models
RPE	Rating of perceived exertion
SMM	Skeletal muscle mass
Sprint	Sprint distance
sRPE	Session*RPE
TD	Total distance
TS	Testing session
