# Supplementary material for: Does Preseason Training Load Shape Body Composition? External Load, Internal Load, and Fatigue in Semi-Professional Football Players
Source: J Funct Morphol Kinesiol. 2026 Oct 6;11(4):410. doi: 10.3390/jfmk11040410 (PMC13692473; doi:10.3390/jfmk11040410)
Supplement: Supplementary file 1 [file jfmk-11-00410-s001.zip › jfmk-4505719-supplementary.pdf]

Supplementary material

Table S1. Weekly evolution of external load-related variables during preseason after excluding goalkeepers.

	Week 1		Week 2		Week 3		Week 4		Week 5		Week 6	
	***Coeff* (*SE*)**	***p***	***Coeff* (*SE*)**	***p***	***Coeff* (*SE*)**	***p***	***Coeff* (*SE*)**	***p***	***Coeff* (*SE*)**	***p***	***Coeff* (*SE*)**	***p***
TD (m)	16467 (1097)	b^{***}, c^{***}, d^{***}, e^{***}, f^{***}	31,269 (1044)	a^{***}, d^{***}, f^{***}	34,725 (1044)	a^{***}, d^{***}, e^{**}, f^{***}	22,471 (1044)	a^{***}, b^{***}, c^{***}, e^{***}	30,156 (1044)	a^{***}, c^{***}, d^{**}, f^{***}	24,345 (1044)	a^{***}, b^{***}, c^{***}, e^{***}
HSR (m)	176.83 (51.01)	c^{***}, d^{***}, e^{***}, f^{***}	312.94 (49.04)	c^{**}, e^{***}, f^{***}	530.78 (49.04)	a^{***}, b^{**}, e^{*}	416.39 (49.04)	a^{***}, e^{**}, f^{*}	686.28 (49.04)	a^{***}, b^{***}, c^{*}, d^{**}	587 (49.04)	a^{***}, b^{***}, d^{*}
Sprint (m)	81.75 (46.63)	b^{*}, c^{***}, d^{***}, e^{***}, f^{***}	207.78 (45.19)	a^{*}, c^{**}, e^{***}, f^{***}	382.67 (45.19)	a^{***}, b^{**}	318.28 (45.19)	a^{***}	421.83 (45.19)	a^{***}, b^{***}	393.5 (45.19)	a^{***}
Sprints (nº)	9.04 (3.49)	c^{***}, d^{***}, e^{***}, f^{***}	16.67 (3.39)	c^{*}, e^{***}, f^{*}	27.17 (3.39)	a^{***}, b^{*}	22.72 (3.39)	a^{***}, e^{*}	33.22 (3.39)	a^{***}, b^{***}	26.22 (3.39)	a^{***}, b^{*}
ACC (nº)	37.89 (6.41)	b^{***}, c^{***}, d^{*}, e^{***}, f^{***}	70.78 (6.22)	a^{***}	67.28 (6.22)	a^{***}	55.94 (6.22)	a^{*}, e^{*}, f^{*}	73.11 (6.22)	a^{***}, d^{*}	74.97 (6.22)	a^{***}, d^{*}
DEC (nº)	35.85 (7.64)	b^{***}, c^{***}, d^{***}, e^{***}, f^{***}	85.5 (7.38)	a^{***}	77.89 (7.38)	a^{***}	87.39 (7.39)	a^{***}	94.39 (7.38)		80.17 (7.38)	a^{***}

Note. Coeff = Coefficient; SE = Standard error; m = meters; TD = Total distance; HSR = High-speed running; Sprint = Sprint distance; ACC = Accelerations; DEC = Decelerations; a = significant different from Week 1; b = significant different from Week 2; c = significant different from Week 3; d = significant different from Week 4; e = significant different from Week 5; f = significant different from Week 6; * $p < 0.05$; ** $p < 0.01$; *** $p < 0.001$.

Table S2. Weekly evolution of internal load-related variables and heart rate during preseason after excluding goalkeepers.

	Week 1		Week 2		Week 3		Week 4		Week 5		Week 6	
	***Coeff* (*SE*)**	***p***	***Coeff* (*SE*)**	***p***	***Coeff* (*SE*)**	***p***	***Coeff* (*SE*)**	***p***	***Coeff* (*SE*)**	***p***	***Coeff* (*SE*)**	***p***
RPE	5.81 (0.25)		6.41 (0.24)		5.84 (0.24)		6.33 (0.24)		5.90 (0.24)		6.47 (0.24)	
RPE*Session	436.94 (24.03)	b*	528.5 (22.77)	a*, c**, f***	399.33 (22.77)	b**, d**	513.07 (22.77)	c**, f***	457.57 (22.77)	f***	355.09 (22.77)	e*
HR 85–90% (min.)	27.51 (4.89)	b*, e*	42.57 (4.72)	a*, d*	38.88 (4.72)		26.50 (4.72)	b*, e*	43.53 (4.72)	a*, f*	29.07 (4.72)	e*
HR 90–95% (min.)	20.29 (4.97)	b*, e*	36.10 (4.80)	a*, d*, f*	30.92 (4.80)		19.13 (4.80)	b*, e*	36.59 (4.80)	a*, d*, f**	17.02 (4.80)	b*, e*
HR 95–100% (min.)	7.67 (2.70)	b*	16.83 (2.59)	a*, d**, f***	13.75 (2.59)	d*, f*	4.92 (2.59)	b**, c*	9.81 (2.59)		3.23 (2.59)	b***, c*

Note. Coeff = Coefficient; SE = Standard error; RPE = Ratio of perceived exertion; HR = Heart rate; min = minutes; a = significant different from Week 1; b = significant different from Week 2; c = significant different from Week 3; d = significant different from Week 4; e = significant different from Week 5; f = significant different from Week 6; * $p < 0.05$; ** $p < 0.01$; *** $p < 0.001$.

Table S3. Weekly evolution of fatigue-related variables during preseason after excluding goalkeepers.

	Week 1		Week 2		Week 3		Week 4		Week 5		Week 6	
	***Coeff* (*SE*)**	***p***	***Coeff* (*SE*)**	***p***	***Coeff* (*SE*)**	***p***	***Coeff* (*SE*)**	***p***	***Coeff* (*SE*)**	***p***	***Coeff* (*SE*)**	***p***
Fatigue	3.71 (0.12)	c*	3.82 (0.11)	c***	3.26 (0.11)	a*, b***, e***, f*	3.52 (0.11)		3.86 (0.11)	c***	3.68 (0.11)	c*
Sleep	3.98 (0.11)	c*	3.93 (0.10)		3.58 (0.10)	a*, e*	3.63 (0.10)		3.99 (0.10)	c*	3.82 (0.10)	
DOMS	3.64 (0.14)		3.67 (0.13)		3.43 (0.13)	e**, f*	3.60 (0.13)		3.94 (0.13)	c**	3.81 (0.13)	c*
Stress	4.23 (0.12)		4.13 (0.11)		3.92 (0.11)		3.96 (0.11)		4.09 (0.11)		4.05 (0.11)	
Mood	4.45 (0.09)	c***, d***, e*	4.29 (0.09)	c**, d*	3.94 (0.09)	a***, b**	4.01 (0.09)	a***, b*	4.01 (0.09)	a*	4.17 (0.09)	
Mental fatigue	4.27 (0.14)	c**, d*	4.00 (0.13)		3.72 (0.13)	a**	3.82 (0.13)	a*	4.00 (0.13)		3.91 (0.13)	

Note. Coeff = Coefficient; SE = Standard error; DOMS = Delayed onset muscle soreness; a = significant different from Week 1; b = significant different from Week 2; c = significant different from Week 3; d = significant different from Week 4; e = significant different from Week 5; f = significant different from Week 6; * $p < 0.05$; ** $p < 0.01$; *** $p < 0.001$.
